# Temporally evolving surround suppression helps decoding in a spiking model of motion processing

**DOI:** 10.1186/1471-2202-14-S1-P188

**Published:** 2013-07-08

**Authors:** Philip M Meier, Micah Richert, Jayram M Nageswaran, Eugene Izhikevich

**Affiliations:** 1 Brain Corporation, San Diego, California 92121, USA

## 

We find that a neural network with temporally evolving surround suppression improves the linear decodability of the network's population response. We present a novel model of motion processing that is fully implemented in a spiking neural network. We examine the role of lateral inhibition in V1 and MT. We model the response of the retina, V1 and MT, where each neuron is a single compartment conductance-based dynamical system. We apply a linear decoder to estimate the speed and direction of optic flow from a population response of a small spatial region in MT.

Before training the decoder on population vector responses from MT with labeled speeds, we allow the spiking neural network to adapt the weights of the recurrent inhibitory neurons with spike-timing dependent plasticity (STDP). This allows the individual cells to adapt their dynamic range to the statistics reflected in the activity of the excitatory feed-forward network. Also, we impose a random onset latency of 1-10 msec for each feed-forward neuron. The combination of the onset latency and the inhibitory STDP results in a surround suppression with a magnitude that modulates throughout the course of the response, balancing the incoming excitatory drive.

The temporally evolving surround suppression affects the activity of excitatory and inhibitory units in V1 and MT. The result is a population response of MT excitatory units that is more informative for decoding. The early response is less direction selective but drives the inhibition that sculpts the later responses. One source of improvement is that inhibition removes the non-selective response, but still preserves a robust selective response. Also the inhibition acts as gain control, which limits how much saturation corrupts the linear code. We measure decoding performance by calculating the sum squared error of an estimate of the direction and speed of optic flow.

## Conclusions

The central question we ask is "Does inhibitory STDP improve the population code of MT, enabling the speed and direction of motion to be better read out by a linear decoder?" We ask this question in a particular context: of having designed a spiking motion processing model that is capable of estimating optic flow, even of small or low contrast stimuli. However, we expect the findings to be general to linear decoding of low SNR inputs into spiking neurons in networks with inhibition. In summary, we find that the answer is, yes, there is a benefit to inhibitory STDP, but only if the onset latencies of the feed-forward neurons are sufficiently staggered in time. Why? The initial volley of spikes engages the lateral connectivity which is capable of predicting the subsequent spikes, and balancing the excitatory drive with inhibition. If the spikes arrive too quickly, there is not enough time to engage the inhibitory machinery.

**Figure 1:**
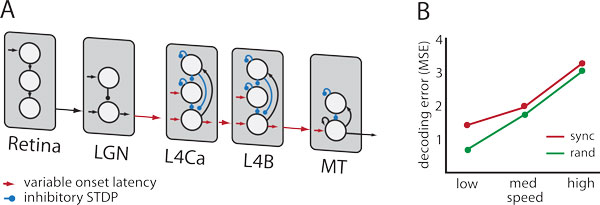
**A) Schematic of model**. B) Model predictions for the speed and direction of a translating object.

